# Patient safety and healthcare quality of U.S. laboratory developed tests (LDTs) in the AI/ML era of precision medicine

**DOI:** 10.3389/fmolb.2024.1407513

**Published:** 2024-08-05

**Authors:** Emma L. Kurnat-Thoma

**Affiliations:** ^1^ Georgetown Institute for Women, Peace and Security, Walsh School of Foreign Service, Georgetown University, Washington, DC, United States; ^2^ Precision Policy Solutions, LLC, Bethesda, MD, United States

**Keywords:** laboratory developed tests (LDTs), FDA proposed rule, CMS clinical laboratory improvement amendments (CLIA), genomic tests, return of results (RoR), regulatory compliance, patient safety, health care quality

## Abstract

This policy brief summarizes current U.S. regulatory considerations for ensuring patient safety and health care quality of genetic/genomic test information for precision medicine in the era of artificial intelligence/machine learning (AI/ML). The critical role of innovative and efficient laboratory developed tests (LDTs) in providing accurate diagnostic genetic/genomic information for U.S. patient- and family-centered healthcare decision-making is significant. However, many LDTs are not fully vetted for sufficient analytic and clinical validity via current FDA and CMS regulatory oversight pathways. The U.S. Centers for Disease Control and Prevention’s Policy Analytical Framework Tool was used to identify the issue, perform a high-level policy analysis, and develop overview recommendations for a bipartisan healthcare policy reform strategy acceptable to diverse precision and systems medicine stakeholders.

## 1 Introduction

Precision and systems medicine increasingly uses patients’ unique lifestyle, environment, genetic and genomic information to make decisions about disease risk and prevention, medical diagnoses and prognosis, therapeutics, and overall treatment plans (Kurnat-Thoma, 2020; Kurnat-Thoma et al., 2020). The National Human Genome Research Institute (NHGRI) defines genetic/genomic testing as the use of an individual’s DNA, RNA, chromosomes, proteins, and metabolites for the purposes of clinically detecting heritable diseases, genotypes, mutations and karyotypes (National Academies of Sciences, Engineering, and Medicine (NASEM), 2017). This information can be used in various ways including risk prediction, disease screening, detection, carrier identification, clinical diagnosis and prognosis in a variety of healthcare, forensics and commercial contexts. Despite the allure of advanced technology, access to safe, affordable, innovative and high quality molecular genetic/genomic tests is a fundamental driver of U.S. healthcare inequity, and carries significant risks including medical errors, unfair patient costs, unjust regional distribution and reimbursement, eugenics and discrimination (Lilley et al., 2022).

**FIGURE 1:**
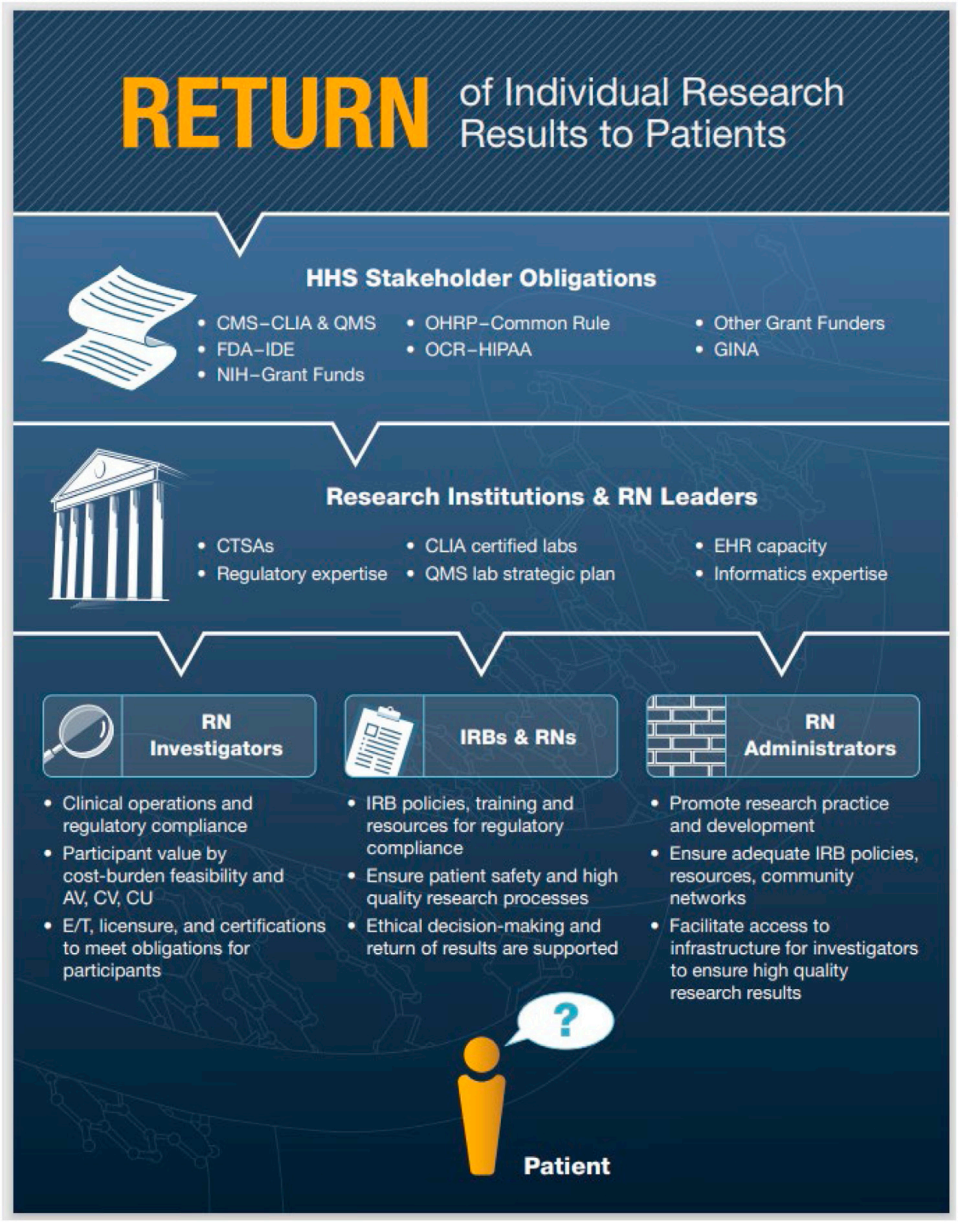
Return of Individual Research Results to Patients. Note. A high level overview of key U.S. regulatory compliance pathways for genetic/genomic RoR for healthcare decision-making with an emphasis on the inclusion of Registered Nurses (RNs) in clinical research workflows (National Academies of Sciences, Engineering, and Medicine (NASEM), 2018). Abbreviations: Return of Results (RoR), Health and Human Services (HHS), Centers for Medicare and Medicaid Services (CMS), Clinical Laboratory Improvement Amendments (CLIA), Quality Management System (QMS), National Institutes of Health (NIH), Food and Drug Administration (FDA), Investigational Device Exemption (IDE), Office for Human Research Protections (OHRP) and 2018 Common Rule Revisions, Office for Civil Rights (OCR), Health Insurance Portability and Accountability Act (HIPAA), Genetic Information Nondiscrimination Act (GINA), Clinical and Translational Science Awards (CTSA), Electronic Health Record (EHR), Analytical Validity (AV), Clinical Validity (CV), Clinical Utility (CU), Education and Training (E/T), Institutional Review Boards (IRBs).

To ensure patient safety, high quality healthcare processes and outcomes, genetic/genomic tests must demonstrate: analytical validity (prediction accuracy, reliability of disease presence or absence); clinical validity (diagnostic sensitivity, specificity, positive, negative predictive values); and clinical utility (improved health outcomes due to test’s use), but are often limited by a lack of sufficient scientific research evidence from randomized controlled trials (RCTs) (National Academies of Sciences, Engineering, and Medicine (NASEM), 2017). Figure 1 summarizes the numerous ethical, legal, social implications (ELSI) in the U.S. when considering the scientific investigation and ethical return of results (RoR) of patients’ genetic/genomic information for healthcare decision-making from clinical research (National Academies of Sciences, Engineering, and Medicine (NASEM), 2018).

For regulatory purposes, laboratory tests are classified as *in vitro* diagnostics (IVDs) or laboratory developed tests (LDTs), or “home brew” tests, with oversight by either Centers for Medicaid and Medicare Services (CMS) or the Food and Drug Administration (FDA) (Budlier and Hubbard, 2023). Most genetic/genomic tests fall under the LDT designation, which meets the medical device definition for FDA oversight. FDA exercises enforcement discretion, which means that most LDTs do not undergo premarket review or receive agency marketing approval clearance, unless the LDT is higher risk, is waived, or explicit guidance is provided during public health emergency periods, such as COVID-19 (Congressional Research Service (CRS), 2020). Common FDA enforcement discretion mechanisms range from informal communications, to publicly posted formal warning letters stating the regulatory violations involved, followed by more serious compliance actions, including criminal enforcement activities (Robinson et al., 2021; Food and Drug Administration (FDA), 2024).

**FIGURE 2:**
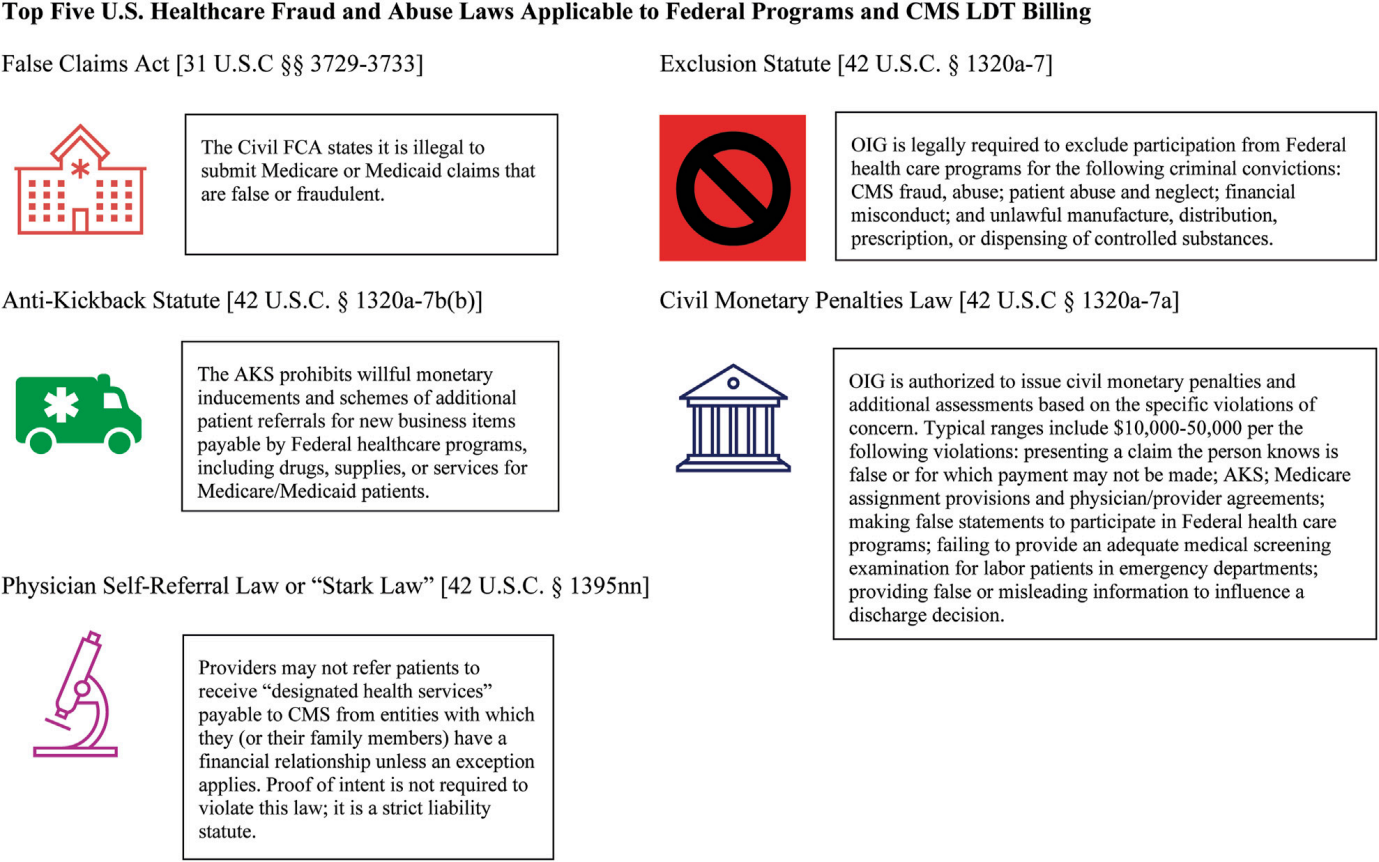
Top five U.S. Healthcare Fraud and Abuse Laws Applicable to Federal Programs and CMS LDT Billing. Note. Summarized here are the top five most important U.S. federal fraud, waste and abuse laws that apply to medical providers involving the use of accurate billing for LDT and related healthcare services as stated by the U.S. DHHS Office of Inspector General (OIG). Consequences of gross violation can result in civil fines, exclusion from Federal healthcare programs, loss of professional licenses from State medical and nursing boards, and criminal penalties (U.S. Department of Health and Human Services (DHHS), Office of the Inspector General, 2024a).

Computational bioinformatics, big data, artificial intelligence and machine learning (AI/ML) approaches have radically altered the algorithm landscape, impacting prediction clinical decision-making accuracy by healthcare providers and clinical decision support system algorithms (U.S. Government Accountability Office (GAO), 2022). For example, erroneous, abusive or fraudulent genetic/genomic/pharmacogenomic (PGx) test information can result in unnecessary care, incorrect therapeutics and dosing, or perpetuate electronic health record errors through multiple payer and provider networks causing profound harms to patients and families, particularly for underserved populations (Sun et al., 2020). CMS defines fraud as knowingly submitting false claims or misrepresentations of fact to obtain federal healthcare payments, and abuse as any practice that directly or indirectly results in unnecessary Medicare program costs that do not meet professionally recognized standards of care (Medicare Learning Network, 2021; U.S. Department of Health and Human Services (DHHS), Office of the Inspector General, 2024a). Priority areas of DHHS Federal program regulatory compliance for LDT use by healthcare providers are highlighted in Figure 2. During the COVID-19 pandemic, long standing frustrations with LDT regulatory oversight and enforcement discretion processes erupted into a serious source of public health concern due to innovation delays, inaccurate tests and exacerbated iniquities, excessive costs due to fraud, regulatory burdens, administrative inefficiency, and ultimately a failed 2022 legislative reform attempt (Marble et al., 2021; Adashi and Cohen, 2022; Dries et al., 2022; Budlier and Hubbard, 2023). Although AI/ML can detect fraud, waste and abuse in novel ways, a key concern moving forward, is diminished level of patient trust in LDTs due to continued historical injustice, lack of knowledge and equity awareness in assay developers, principal investigators and healthcare staff, lack of full inclusion in genomic databases, regulatory loopholes, and diminished community involvement (Medicare Learning Network, 2021; Lilley et al., 2022; Suesserman et al., 2023; U.S. Department of Health and Human Services (DHHS), Office of the Inspector General, 2024a, 2024b).

Thus, this policy brief asks the following question: how can the U.S. ensure safe, high quality patient- and family-centered precision medicine using innovative genetic/genomic information from LDTs in the artificial intelligence/machine learning (AI/ML) era?

## 2 Methods

The Centers for Disease Control and Prevention (CDC) Policy Analytical Framework Tool was used to identify the issue, analyze publicly available documents and relevant scientific evidence, identify policy options and help develop a federal strategy for furthering a final solution (Centers for Disease Control and Prevention (CDC), 2022a). In addition to seminal legacy reports, a PubMed narrative review for peer review literature in 2010–2024, helped inform a precision medicine regulatory landscape using the focused key terms: *in vitro* diagnostics (IVD), laboratory developed tests (LDTs), CMS Clinical Laboratory Improvement Amendments (CLIA), FDA and enforcement discretion. Government databases were queried for information pertaining to: voluntary genetic test information (Gene Tests, FDA-CLIA); legislative bill status (Congress.gov); and grey literature reports on LDTs, CLIA and FDA genetic test regulatory safety, quality oversight (i.e., Congressional Research Service (CRS), U.S. Governmental Accountability Office (GAO), Biden/Harris Administration National Strategy Documents, President’s Council of Advisors on Science and Technology, etc.).

All LDT content was included regardless of clinical specialty using NHGRI’s operationalized definition of a genetic/genomic test (National Academies of Sciences, Engineering, and Medicine (NASEM), 2017). Direct-to-consumer, forensics and identity genetic testing unrelated to an established healthcare provider were excluded. Non peer-reviewed sources—supplemental news analyses, non-profit agency, think tank reports, draft legislation and rules, electronic Code of Federal Regulations, and other medical editorial sources were also identified, since they are used by stakeholders to inform bipartisan legislative debates and clarify contentious federal oversight issues. Including these policy documents helps to understand regulatory barriers encountered in the commercial healthcare sector and to gauge bipartisan political, operational feasibility for moving forward. Although not considered “direct scientific evidence” (i.e., clinical trial research), failing to take the full scope of these policy concerns into serious consideration ultimately impacts Congressional Committee, Sub-Committee votes and increases the likelihood of political instability in the form of: increased corrective oversight through executive orders, declined bills, increased Congressional letters to federal agencies, and costly judicial branch court challenges for specific federal agencies.

## 3 Policy insights

### 3.1 Genomic return of results (RoR) to patients and families

Once the Human Genome Project and additional subsequent federal sequencing initiatives were made publicly available, it was widely assumed this knowledge would result in improved patient safety and health care quality outcomes via tailored prevention, diagnosis and therapeutics to help improve the health of patients and families from diverse communities and ethnic subpopulations (U.S. Department of Health and Human Services (DHHS), 2008; Ferreria-Gonzalez et al., 2008; Fu et al., 2020). For example, the American College of Medical Genetics (ACMG) annually releases a policy statement supporting RoR of pathogenic or likely pathogenic secondary findings, to patients and families unrelated to the original presenting clinical complaint, from whole genome sequencing (WGS) and whole exome sequencing (WES) (National Academies of Sciences, Engineering, and Medicine (NASEM), 2018; Miller et al., 2023). However, the complexity and pace of next-generation sequencing (NGS), -omic and AI/ML technologies grows annually without commensurate implementation capacity and workforce knowledge in many clinical settings, fostering reimbursement conditions ripe for neglect, discrimination, misuse, abuse, waste, fraud, and even financial kickback schemes in excess of $2.1 billion (National Academies of Sciences, Engineering, and Medicine (NASEM), 2018; U.S. Department of Justice (DOJ), 2019; Kurnat-Thoma, 2020; Kurnat-Thoma et al., 2020; Sun et al., 2020; DHHS, U.S. Department of Health and Human Services (DHHS), Office of the Inspector General, 2024a, 2024b). Polygenic risk score predictions, where genomic variant information is used to predict future clinical risk of complex heritable traits or diseases, are especially difficult to provide algorithmic transparency due to rapid technology changes and interpretation significance in the context of population-based profiling (O’Sullivan et al., 2022; Food and Drug Administration (FDA), 2023). Currently, genomic RoR for complex conditions using AI/ML for polygenic risk score predictions, occurs in intensive investigational research contexts, such as the eMERGE program (Linder et al., 2023).

### 3.2 Patient safety and healthcare quality

Prioritization of patient safety and healthcare quality are foundational cornerstones of U.S. healthcare systems research since the publication of two landmark Institute of Medicine reports: To Err is Human (2000) and Crossing the Quality Chasm (2001). A new healthcare vision was developed, and quickly expanded to a global scale for the critically needed monitoring of stark deficits across six healthcare dimensions: safety, timeliness, patient- and family-centeredness, effectiveness, efficiency and equity (Berwick et al., 2018). Patient- and family-centered health care prioritizes mutual respect, trust-based, two-way communicative partnerships between patients, families and their health care providers, to achieve the desired values of improved patient safety, clinical effectiveness, health outcomes and decreased costs; it is an affirmed approach in medicine and nursing ethics for interdisciplinary collaboration (Clay and Parsh, 2016; Millenson et al., 2016).

Recognizing the continued severity of high rates of dangerous adverse events, the President’s Council of Advisors on Science and Technology (PCAST) recently launched a Transformational Effort on Patient Safety. This initiative prioritizes a robust U.S. national safety science research enterprise capable of directly addressing unsafe healthcare and systems level-approaches and failures, including but not limited to: diagnostic errors, medical device malfunctions, medication and surgical errors, failure to rescue, patient injuries and never events (President’s Council of Advisors on Science and Technology (PCAST), 2023). Never events are particularly shocking and completely preventable medical errors that should never occur, and result in serious disability, disappearance, or death of the patient and/or family members (Agency for Healthcare Research and Quality (AHRQ), 2019). In 2018, 25% of all CMS patients experienced harm in hospitals, with a quarter of these patients having to pay additional Medicare costs; nurse and physician reviewers determined almost half were preventable (43%) and were related to delirium and altered mental status from medications/therapeutics (U.S. Department of Health and Human Services (DHHS), Office of the Inspector General, 2022). In 2015, a National Academies report identified misdiagnosis accounts for 10% of patient deaths and 17% of hospital adverse events (National Academies of Sciences, Engineering, and Medicine (NASEM), 2015). Diagnostic errors are commonly directly reported by patients and families; an estimated ∼795,000 Americans are permanently disabled for the remainder of their lives or die annually in the U.S. healthcare system due to catastrophic misdiagnosis of dangerous diseases, increasingly involving use of genomic information (i.e., NGS identification of infectious diseases, cancers, etc.) (Newman-Toker et al., 2023; President’s Council of Advisors on Science and Technology (PCAST), 2023).

For precision and systems medicine, many operational areas of patient safety and healthcare quality outcomes measurement in clinical operations are underdeveloped or absent; for which open access healthcare data can readily be analyzed by journalists and media, followed by constituent letters to policymakers and agency safety communication notices (Korngiebel et al., 2016; Kurnat-Thoma et al., 2021; Food and Drug Administration (FDA), 2022). Korngiebel et al.’s (2016) pilot qualitative study summarized several patient safety concerns by genomic medicine key informants. This study featured an anecdotal report of a Huntington Disease patient committing suicide after receiving their terminal diagnosis over the phone. Unfortunately, this was not recognized by their medical providers as an avoidable “never event” necessitating additional patient safety protection or care management support. The same study also identified numerous genetic testing adverse events including: patients and families failing to receive adequate support when receiving sensitive and traumatic information, medical and ancillary staff having poor knowledge about which tests to order resulting in significant out of pocket costs, incorrect provider ordering errors, patient lab sample mix-ups, unnecessary life-altering surgeries, excessive community criticism for accurate documentation to ensure correct test interpretation and insurance reimbursement, etc.

Summarily, this portends difficulty for introducing advanced WGS/WES and AI/ML technologies in widespread clinical settings without sufficient clinical research and laboratory quality management infrastructure, patient- and family-centered diagnostic testing engineering, human factors, implementation science, workforce development, health care quality innovation, patient safety reporting and adverse event surveillance.

### 3.3 VALID act

Due to their profound impact on individual patient, family, community and public health, accurate laboratory diagnostics play a critical and irreplaceable role for U.S. healthcare. Every year, approximately 70% of U.S. medical decisions depend on a total ∼14 billion laboratory tests across ∼330,000 CLIA-certified laboratories (Centers for Disease Control and Prevention (CDC), 2018; 2022b). Disruptions to this fragile system, especially in rural geography and/or poorly resourced systems, contributes to ∼40,000–80,000 preventable American deaths per year due to diagnostic errors, leaving our national security at unacceptable levels of heightened risk from harmful false positive and negative results during infectious disease outbreaks, bioterrorist, chemical and radiologic threats (Centers for Disease Control and Prevention (CDC), 2018; 2022b). False negative results cause the disease to progress undetected without opportunity for life-saving treatment. False positive results cause inappropriate and invasive treatment, additional costs, serious irreversible harms, delays the true diagnosis, and results in significant anxiety and distress (Food and Drug Administration (FDA), 2023).

Most genetic/genomic tests are LDTs, a category of IVD that are designed, produced and utilized within a single laboratory, and proceed to market without independent analysis and verification of the information provided (Congressional Research Service (CRS), 2022; National Human Genome Research Institute (NHGRI), 2024). FDA regulates the safety, effectiveness, design quality and manufacturing of moderate and higher risk genetic/genomic tests (analytical and clinical validity). In 1976, the U.S. Congress granted oversight authority to FDA for Medical Device Amendments under the Federal Food, Drug, and Cosmetic Act (FD&C Act). At the time, LDTs were simple manual assays without automation using straightforward components and analytes, thus FDA uses “enforcement discretion”—the agency chooses not to use the regulatory authority granted it. FDA guidance documents present additional clarity on the agency’s LDT enforcement discretion framework. Complex genetic/genomic LDT tests, kits are categorized as medical devices (Investigational Device Exemption [IDE]) and a risk-based determination is used (Class I general controls, Class II special controls, or Class III premarket approval), where moderate or high level of risk categories require general and special controls, pre-market approvals and adverse reporting to the agency (Food and Drug Administration (FDA), 2014; Robinson et al., 2021). These requirements often have varying regulatory implications based on the particular technology involved required for specific clinical applications and sub-populations such as oncology, genetics/genomics, microbiology, or other omics for biomarker, proteomics, and metabolite detection.

In parallel to FDA, CMS’ CLIA program regulates LDT use in clinical laboratories by specifying quality standards and testing performance thresholds for accurate, reliable results on patient specimens used for health care decision making (analytical validity), primarily in the form of accreditation, certification, proficiency testing, and biennial inspections from CMS or an independent entity with deemed status (Budlier and Hubbard, 2023; Centers for Medicare and Medicaid Services (CMS), 2024). In 1988, the U.S. Congress enacted significant reforms to the CMS CLIA program by strengthening laboratory quality assurance certification measures after the Wall Street Journal published a 1987 analysis of profound failures of Pap smear testing outcomes due to a poorly trained and unsupervised workforce (Graden et al., 2021). Historically, FDA and CLIA work complementarily on making regulatory determinations for IVD device manufacturers regarding categorization of waived, moderate complexity and high complexity tests (Food and Drug Administration (FDA), 2017). CLIA laboratories performing the highest complexity testing must maintain stricter workforce education, training, certification and accreditation requirements.

**FIGURE 3:**
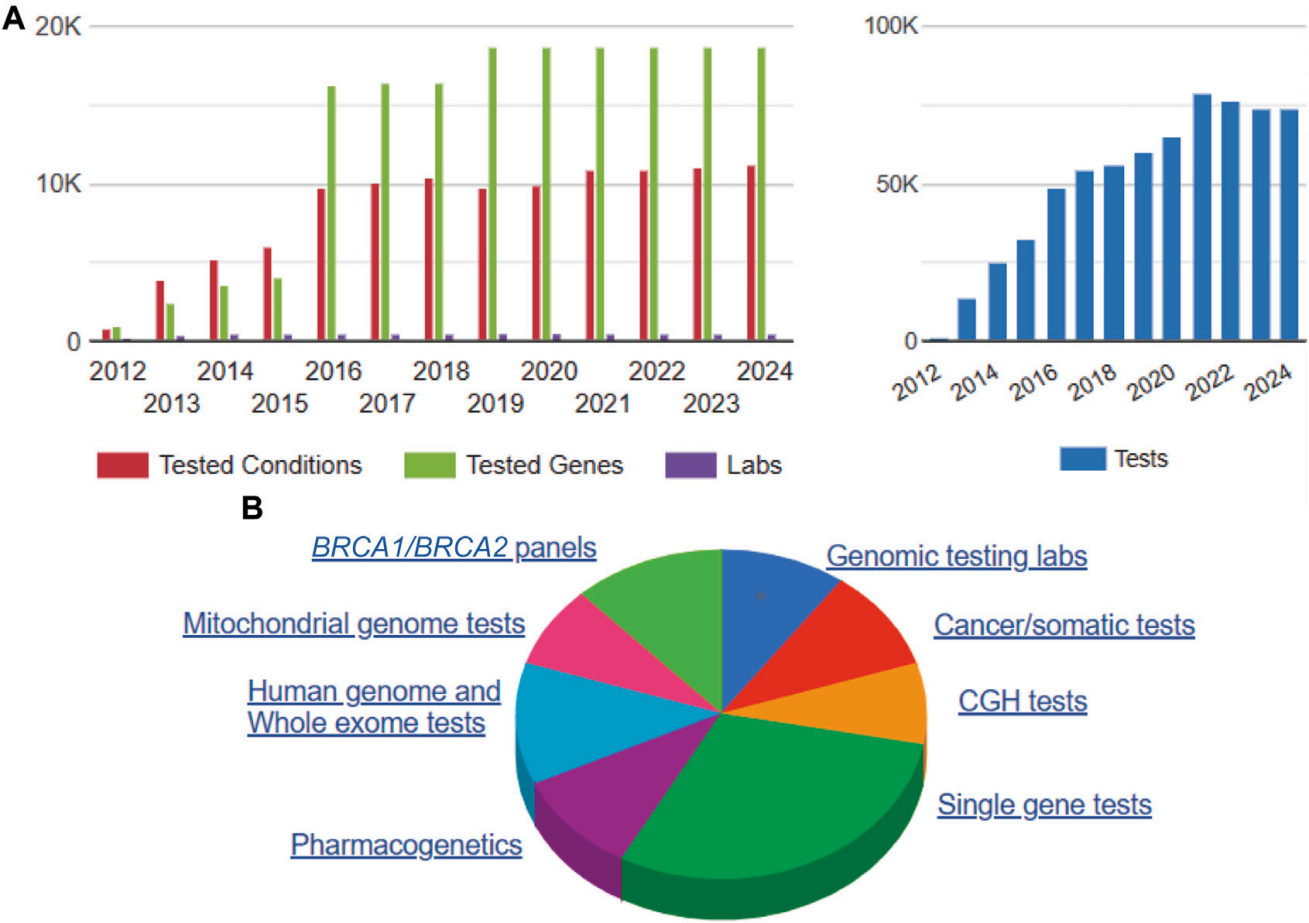
NCBI Annual Genetic Testing Registry Data 2012–2024. Note. NCBI 2012–2024 GTR data comprises annual voluntary submitted (non-validated) genetic/genomic test registration information by lab, genes, conditions and test type (National Center for Biotechnology Information (NCBI), 2024). Summary information on 5/10/2024 includes: 72,980 genetic/genomic tests for 25,413 disease conditions and 18,703 genes from 447 laboratories **(A)**. Of the total voluntarily submitted genetic/genomic test information, more than half are single gene tests, WGS/WES, and pharmacogenetics tests, which are likely to involve AI/ML applications for risk prediction and clinical decision support systems. **(B)**. Abbreviations: Comparative Genomic Hybridization (CGH), *BRCA1/BRCA2* breast cancer gene test panels.

As shown in Figure 3, voluntary self-reported data reported to the National Center for Biotechnology Information (NCBI) Genetic Testing Registry (GTR) indicates an annual trend of increasing genetic/genomic testing volume and complexity (i.e., WES/WGS). Although registry information is not agency-verified for accuracy, it is a helpful general resource for monitoring overall growth trends, including CLIA certification and FDA review status (Horrow and Kesselheim, 2024; National Center for Biotechnology Information (NCBI), 2024). However, in the era of AI/ML applications, LDTs are used in more sophisticated and complex ways involving multi-component assay kits, sequencing systems, software, algorithms, and complex, sensitive instrumentation with little transparency or accountability for quality, particularly in the form of adverse events and safety information (U.S. Government Accountability Office (GAO), 2017; U.S. Government Accountability Office (GAO), 2022; Food and Drug Administration (FDA), 2023). Markets strategically select these innovation areas to predict likelihood of serious medical conditions such as cancer, infectious diseases, and heart disease due their high value, and thus more LDTs are used in the clinic without full evaluation of analytic and clinical validity (National Human Genome Research Institute (NHGRI), 2024). Review of multi-sourced literature indicates an increasing trend problem. There are needless patient deaths, injuries and adverse events, workforce deficiencies, lost productivity and increased healthcare costs from erroneous, inaccurate medical diagnostics, involving the use of LDTs with particular implications for high need areas for clinical off-label scenarios including: prenatal care, inborn errors of metabolism, pediatrics, pharmacogenomics, cancer, infectious diseases and pandemic preparedness (Food and Drug Administration (FDA), 2015; Korngiebel et al., 2016; Pew Charitable Trust, 2021; Food and Drug Administration (FDA), 2023; 2022; Bunch, 2023; President’s Council of Advisors on Science and Technology (PCAST), 2023; Miller, 2024).

Patient harms and consequences are increasingly serious and span cancer, prenatal conditions and pregnancy care, detection of rare genetic mutations, pharmacogenomic dosing and inappropriate companion diagnostic therapies, and inability to rapidly respond to emerging infectious disease public health outbreaks. Scientific literature, news articles reporting results from independent analyses, class action lawsuits, consistent bipartisan congressional inquiries, and profound high-profile failures identified by investigative journalism teams (i.e., corporate scandals for fraudulent tests, non-invasive prenatal genetic testing false positives, etc.) raises serious questions if Americans can trust their diagnostic tests and healthcare decision-making due to exploitation of regulatory gaps and loopholes by bad actors seeking financial gains. The Verifying Accurate Leading-edge *in vitro* clinical test Development Act (VALID) introduced to Congress in 2018–2020 proposed to create a new regulatory pathway for *in vitro* clinical test kits and LDTs, and clearly define scope of FDA authority for review and approval, but this did not pass the Senate in 2022 (Graden et al., 2021; Budlier and Hubbard, 2023; Stone and Gugten, 2023). In response to this failed legislation, in September 2023 the FDA proposed a new rule for LDT oversight which is generating concern for small-medium size laboratories unable to meet the demands of additional regulatory requirements (Genzen, 2019, p. 125; Food and Drug Administration (FDA), 2023).

### 3.4 Select AI/ML Biden/Harris administration initiatives

In addition to PCAST, several Presidential Executive Orders in the Biden/Harris administration and federal agency strategic initiatives are driving accelerated AI/ML research and development (Bipartisan Policy Center, 2023). Cumulatively, these have implications for how science, medicine and healthcare communities can proactively navigate agency rulemaking, legislation efforts. The Blueprint for an AI Bill of Rights stipulates five anchor protections that all Americans are entitled to during the design, development and implementation of innovative AI/ML technology systems and medical devices, which are especially relevant for accurate healthcare and diagnostic tests using genetic/genomic information (The White House, 2022a). For example, the U.S. Equal Employment Opportunity Commission (EEOC) Office for Civil Rights, Diversity, and Inclusion recognizes equity “without regard to race, color, sex, religion, national origin, age, disability, sexual orientation, genetic information or reprisal” (Equal Employment Opportunity Commission (EEOC), 2024). When scientists, providers, manufacturers-developers, and federal agencies design, use, review and approve LDTs with genetic/genomic information, all Americans should be: 1) protected from unsafe and/or ineffective systems; 2) protected from algorithmic discrimination and outcome inequity based on any legally recognized classification (i.e., Equal Employment Opportunity Commission (EEOC), 2024); 3) be ensured of data privacy and understand how personal data is used for outcomes, and any relevant civil liberties contexts; 4) understand and know how private data is serially propagated and used throughout multiple systems and networks; and 5) have recourse to human alternatives, consideration and fallbacks to troubleshoot especially harmful errors through the use of a clearly stated appeal or escalation process, access lawfully available civil protections mechanisms, and be granted the right to drop out due to excessive harms without any opportunity for direct human interaction. The Blueprint for an AI Bill of Rights is a powerful policy cornerstone for the ethical, legal design of scientific research advancements and regulatory oversights that can genuinely improve human progress and wellbeing, while mitigating serious bias harms from misuse and abuse without careful consideration.

Several Biden/Harris Administration initiatives feature use of AI/ML to ensure accurate diagnostics for a safe, high quality healthcare system to ensure national preparedness for future pandemics, infectious disease outbreaks, bioterrorism and to counter biological threats with rapid responsiveness of healthcare, laboratory supply chains, acquisitions and procurements, public health, research and development infrastructure coordination (The White House, 2022b; 2023; Department of Defense (DOD), 2023; Executive Office of the President, 2023; Bipartisan Policy Center, 2023). Additionally, as of 18 March 2024, a query of the Biden/Harris White House Administration Statements and Releases for ‘artificial intelligence’ yields ∼400 public press briefings spanning all domains of American commerce, scientific research, development, and public life (The White House, 2024b). Specific priorities and themes driving the U.S. AI/ML ecosystem impacting accurate genetic/genomic LDTs in healthcare include: responsible innovation; thriving economies and new jobs with equitable distribution of technological benefits; safe and responsible use; secure and trustworthy development and use; protection of law, civil rights, and the interests of consumers and workers; fair market competition and small businesses; inclusive STEM education; workforce development and diversity; secure infrastructure and cybersecurity; adequate protections from malicious actors, theft and crime; privacy protection and individual rights; environmental sustainability and sustainable development; unparalleled scientific and technical expertise with safety and trust; shared prosperity and value.

## 4 Proposed recommendations

After reviewing the evidence and considering current feedback in the published literature, three overall policy strategies are possible moving forward: 1) optimize FDA LDT regulation in the proposed rule; 2) modernize CMS CLIA; 3) maintain the current status quo. Table 1 presents a high-level summary overview of policy recommendations and their advantages and disadvantages.

**TABLE 1:** Policy recommendations for strengthening U.S. Regulatory oversight of LDTs.

Policy recommendation	Implementation strategy pros	Implementation strategy cons
Optimize FDA Enforcement Discretion	• Is a traditionally recommended approach	• High complexity tests are the most lucrative, which increases likelihood of misconduct, fraud, waste and abuse
	• Well-funded large urban cities with improved access to high quality, safe LDTs, Companion Diagnostics (CDx)	• FDA addresses analytical and clinical validity in the pre-marketing clearance and approval process, and readily identifies fatal methodologic flaws which can be costly
	• Large academic medical centers and commercial laboratories have strong incentive for prioritizing omics LDT innovation d/t available grant, patent/intellectual property/legal infrastructure pipelines	• Costs favor large well-funded institutions that are administratively endowed for multi-sector expertise and the capacity to correctly respond to agency methodological concerns (i.e., Figure 1)
	• Is a profitable area of scientific research	• Smaller, less resourced hospitals, AMCs with high quality laboratory developed procedures (LDPs) under CLIA cannot easily add FDA regulatory requirements and keep costs manageable, will remove LDTs
	• FDA 510(k) and PMA IVD pipelines are well established and can be scaled up	• Proposed 4–5 years enforcement discretion phaseout is unrealistic for small, medium labs
	• FDA estimates $2.67–86.01 billion in savings over 20 years from direct health benefits from avoidance of faulty LDTs	• Increased likelihood of increased financial costs due to CMS health insurance claim rejection for NGS genetic/genomic tests, which requires additional patient payment assistance programs
	• Proposed rule includes transparent LDT adverse event reporting, correction and removal to monitor severe harms	• Small towns, middle, rural America would experience exacerbated resource gaps and U.S. healthcare disparities
	• Requires prospective data from clinical trials	• Costs, delays, LDT gaps would decrease capacity, inequity in key policy areas including maternal and perinatal healthcare, opioid abuse crises, and oncology and infectious disease
	• Increased likelihood of CMS financial reimbursement for medical devices, CDx and PGx drugs in Medicare payments	
Modernize CLIA	• Focuses on assuring high quality laboratory processes, procedures for LDT use in clinical laboratories	• Evaluates LDT lab processes after they are in clinical use; may not always use informed consent
	• Can add clinical validity for designated categories, and harmonize with FDA	• CMS has traditionally preferred FDA to handle clinical validity for LDT regulatory oversight
	• CMS’ CLIA is a preferred regulatory structure for ensuring flexibility for off-label applications in routine clinical settings	• Permissive and heterogeneous operating structure such that underqualified doctoral personnel (i.e., 1 year of lab experience) can easily set up their own LDTs without fully understanding the consequences of insufficient formal evaluation
	• Reimbursement and payment model is a more comfortable language compared to FDA for clinical providers	• Analytical validation limited to the conditions of the laboratory, staff, equipment, and patient populations
	• Endorsed by specialty laboratory professional groups and multi-sector stakeholders such as Association of Molecular Pathology (AMP), and the American College of Medical Genetics (ACMG) with a draft legislative proposal	• Requires a knowledgeable and qualified medical director to appropriately hire, direct the specialized staff for the complexity level, scope, coverage for the particular LDT or advanced technology involved
	• Additional requirements can be designed by the laboratory science community for current accreditation and proficiency testing structures that are harmonized with FDA.	• Increased costs of proficiency testing, accreditation requirements will decrease LDT availability and impact patient operations
	• Can add regional peer review mechanisms for high-complexity laboratory directors to strengthen or stratify specific regulatory layers by LDT technology or method (i.e., oncology [liquid biopsies], NGS, qPCR, MALDI-TOF, LC-MS/MS assay, prenatal testing, AI/ML, etc.)	• If no CMS benchmarks, need a method for reporting LDT-related adverse events for safety and quality monitoring transparency
	• Can link to CMS benchmarks for improved laboratory accountability	• Medicare/Medicaid (CMS) fraud, waste and abuse is a persistent challenge, especially in emerging scientific innovation areas of great need (i.e., COVID-19)
Maintain Status Quo	• Allows industry to self-regulate within their preferred and current cost, operational and regulatory resource constraints	• Range of LDT complexity and no formal mechanism to track genomic testing numbers, complexity, types of testing services, means it will not be possible to understand the worst healthcare disparities, patient and family harms
	• FDA’s voluntary CDx pilot program offers co-development of oncology drug products with certain types of LDTs (NGS, PCR, FISH, IHC) linked to an IVD structure	• Continued lack of transparency for the most egregious harms, due to no requirements for publicly reporting LDT-related adverse events to some sort of central authority
	• Can revise current databases such as FDA-CLIA, FDA Manufacturer and User Facility Device Experience (MAUDE), FDA Medical Device Recalls for strengthened adverse event measures and data visualization	• Large-scale LDT modifications for tests with complex components in CLIA labs, followed by promotional marketing as FDA-alternatives, are not easily distinguishable from truly FDA-vetted tests
	• Optimize NCBI Genetic Testing Registry, for additional voluntary self-submitted information about relevant LDT safety, quality, adverse events, AI/ML and clinical decision support system use, FDA review and CLIA certification status components	• Fragmented, non-harmonized regulatory frameworks means continued litigation for patient harms and medication, diagnostic errors
	• Permissive regulatory environment of FDA enforcement discretion means most LDTs are unregulated and maximally favoring local autonomy and finance models	• No informed consent, adverse reporting, clinical validity in CMS CLIA
	• CMS-CLIA certification for high complexity laboratories can provide LDT informed consent if using DHHS Common Rule before the tests are used on patients	• CMS CLIA surveys indicate significant laboratory challenges, deficiencies in workforce qualifications and non-compliance with manufacturer instructions
	• Consider development and addition of LDT error rates, never events, adverse events to AHRQ Quality and Patient Safety Indicators	• Anticipate continued independent journalism analyses of corporate scandals, LDT failures, health care quality, patient safety issues and implications (i.e., COVID-19), followed by media and policy stakeholder scrutiny and agency accountability
		• Older adults and vulnerable, underserved populations are especially susceptible to genetic/genomic testing fraud, misuse and abuse schemes linked to illegal kickbacks, particularly in the areas of prenatal screening and cancer screening, diagnosis, treatment
		• Can have adverse impacts on the level of public trust in U.S. science, public health and healthcare infrastructure

### 4.1 Policy option 1: optimize FDA LDT rulemaking

Failed VALID Act legislation and the newly proposed FDA rule for LDT oversight has generated significant concern from a variety of stakeholders in the laboratory community about excessive costs, regulatory burdens and innovation barriers depending on the type and complexity of the particular tests and diseases involved, and identified possible optimization pathways for complementary agency roles (Graden et al., 2021; Offit et al., 2022; Food and Drug Administration (FDA), 2023; Miller et al., 2024; Horrow and Kesselheim, 2024). For example, some LDTs receive greater regulatory scrutiny via the CMS CLIA accreditation, proficiency testing, and biennial inspections than the FDA-approved tests used by the same laboratory, or involve specific applications that require significant flexibility for rapid turnaround times (U.S. Government Accountability Office (GAO), 2017; Geno and Cervinski, 2023; Miller et al., 2024). There is also significant lack of clarity of regulatory jurisdiction between FDA IDE, DHHS Common Rule (45CFR46) Institutional Review Board informed consent requirements, and CMS CLIA requirements for high complexity laboratories when using NGS in investigational research (Figure 1); this may represent an area for regulatory optimization especially since some states use more strict criteria (National Academies of Sciences, Engineering, and Medicine (NASEM), 2018; Venner et al., 2022; Wolf and Green, 2023, p. 402). Adequate workforce development, education and training, technical proficiency competency are common themes throughout most reports.

Although there is a need for accuracy, safety, and utility, an optimal balance must be struck for time and complexity requirements, staffing burdens, and concomitant costs, especially for smaller laboratories with vulnerable patients needing prompt healthcare answers for reasonably accurate decision-making in life-death circumstances (Bunch, 2023). Downstream implications of intensive regulatory frameworks have profound impacts on laboratories, clinicians and patients, for which the recent COVID-19 pandemic both highlighted powerful opportunities for flexibility, innovative change and vulnerable failure points (Marble et al., 2021; Budlier and Hubbard, 2023). Failure to fully consider the legitimate concerns of smaller laboratories, clinics, hospitals, practitioners and vulnerable/underserved populations will increase healthcare disparities in precision medicine. It will also result in pendulum-style retaliatory legislative corrections, payment coverage decisions, and judicial challenges of aggressive research and development agendas for grants, research and development financial gains, intellectual property, and patents and increase the likelihood for fraud, waste, and abuse (Klein, 2020). Because some LDTs fall only under the practice of clinical medicine, at the very worst, it could be duplicative regulation if clinical laboratory tests overlap with commercial manufacturing operations, especially if designed without harmonization of bipartisan policy stakeholder feedback and reviewing previous regulatory, judicial precedent pain points (Thompson et al., 2016; Bipartisan Policy Center, 2023). Continued genetic/genomic testing waste, fraud and abuse is also a significant area of concern given the ecosystem of increasing financial rewards associated with successful market innovations (U.S. Department of Justice (DOJ), 2019; Sun et al., 2020; U.S. Department of Health and Human Services (DHHS), Office of the Inspector General, 2024b). The one published peer review report available on FDA’s proposed rule highlights concerns regarding public health microbiology LDT innovation, responsiveness and turnaround times that would negatively impact health infectious disease diagnostics, healthcare access and equity, and projects significant limitations from the 4-year enforcement discretion phaseout implementation timeline plan, compared to the VALID Act’s plan of 9 years (Miller et al., 2024).

### 4.2 Policy option 2: modernize CLIA legislation

CLIA has not been significantly modified since 1988, before the initiation of the Human Genome Project. In addition to strengthening FDA LDT oversight, multiple scholars make the case for also modernizing CLIA to include strengthened analytical and clinical validity coverage due to frequent FDA modifications are cost and time prohibitive for staff, even for high complexity laboratories that process WES/WGS (Kaul et al., 2017). The Association for Molecular Pathology (AMP) drafted a CLIA modernization legislative proposal that expands various definitions and criteria, particularly for analytical and clinical validity in the context of NGS, and has the support of over 50 multisectoral policy stakeholders inclusive of commercial industry (Association for Molecular Pathology (AMP), 2023). The AMP proposes a more flexible oversight system that can better monitor the accuracy and quality of more advanced laboratory developed procedures (LDPs) while ensuring transparency and continued innovation (Association for Molecular Pathology (AMP), 2023). An optimized CLIA regulatory model for precision medicine components can allow for improved proficiency testing flexibility and the innovative development methods that are especially needed during public health emergencies involving nucleic acid sequencing (i.e., COVID-19 and/or other rapidly emerging public health threat). It would also work with CMS deemed status entities such as the College of American Pathologists, New York State Dept of Health and others. Examples include: NGS and quantitative PCR assays in genomics/oncology, companion diagnostics for pharmacogenetics (CDx and PGx), Matrix Assisted Laser Desorption Ionization-Time of Flight (MALDI-TOF) in clinical microbiology, and a wide variety of mass spectroscopy-based methods in clinical chemistry for metabolomics, proteomics, and innovation opportunities for strengthening precision, turn-around times for critically needed patient information, while avoiding the lengthy FDA review process (Kaul et al., 2017; Lin and Thomas, 2023).

### 4.3 Policy option 3: maintain status quo by strengthening safety and quality outcome data

In all literature and agency documents, it is widely recognized that both CLIA and FDA frameworks could be harmonized, improved and/or modernized to better meet the needs of the full spectrum of laboratory science, medical, healthcare communities, policy and public stakeholders that directly depend on quality oversight integrity. This was also an important consensus conclusion from a pre-pandemic expert advisory committee investigating how to address the regulatory oversight gaps and loopholes for return of results of genetic/genomic sequencing and other complex health information to patients and families (National Academies of Sciences, Engineering, and Medicine (NASEM), 2018). However, there does not seem to be sufficient political will or consensus on preferred paths to accomplish that given the complexity and monies involved, such that some leaders opine “something drastic has to occur” before the issue is taken seriously enough to pass Congressional legislation that adequately protects patients and families to ensure accurate diagnostics.

Without further legislative modification of either FDA LDT oversight or CLIA, CMS will continue to use its enforcement discretion capacity to limit Medicare financial coverage for provider use of precision medicine drugs and medical devices without sufficient scientific evidence from clinical trials or registries, due to they are not “reasonable or necessary” (Daval and Kesselheim, 2023). Common examples are “coverage with evidence development” for warfarin pharmacogenomic testing, and “covered with conditions” for NGS tests in beneficiaries with designated advanced cancers. Thus, an essential component to maintaining a strong post-COVID-19 recovery, is sufficient investment in scientific research and development for both basic and clinical research especially in underserved populations, including older adults, women, children, racial and ethnic minorities who greatly need accurate genetic/genomic test information and efficient turn-around times for results (The White House, 2024a). This emphasis must also include both safety and quality for multiple scientific domains from which precision medicine LDTs are developed and used in healthcare (Fauci and Folkers, 2023; Agency for Healthcare Research and Quality (AHRQ), 2024a, 2024b). Ensuring adequate knowledge and clinical research infrastructure that is immediately available to detect and respond to severe biological threats and/or pathogens that can greatly harm the American people must remain an omnipresent and constant concern (Fauci and Folkers, 2023).

In the absence of modernization legislation and/or final FDA rule implementation, modest changes can be considered to better link the worlds of fraud detection and monitoring, medical device, laboratory and hospital safety and healthcare quality for strengthened transparency and accountability. The top CLIA deficiencies in the U.S. in 2021 were for the personnel competency assessment standard (*n* = 726, 18.96%), followed by analytic systems in accordance with manufacturer instructions (*n* = 670, 17.50%), and biennial general lab systems measurement procedures (*n* = 623, 16.27%) (Centers for Medicare and Medicaid Services (CMS), 2021). A recent federal investigation by the Department of Justice of genetic testing fraud in 2018 identified over 840 clinical laboratories providing genetic testing in 45 states, with most claims comprising single gene procedure codes (62%), multi-analyte assays with algorithmic analyses for cancer (18.4%), and non-invasive prenatal testing (2.6%), multiple gene tests (2.0%) and WGS (1.8%) (Sun et al., 2020). Current NCBI Genetic Testing Registry, CMS hospital surveys, and Agency for Healthcare Research and Quality (AHRQ) healthcare quality and patient safety indicators do not include use of AI/ML, diagnostic testing related errors, never events or adverse events, so these would need to be carefully considered for where transparent LDT adverse event information would best fit without being redundant. AI/ML algorithmic clinical risk predictions should be clearly differentiated from laboratory and organizational data analytics for aggregate benchmarks. A number of current FDA resources could be strengthened to better track outcome data for medical device adverse events, never events, or serious malfunctions such as the databases for medical device recalls and manufacturer and user facility device experience (MAUDE) resource. For example, due to the increasing complexity of AI/ML components, it may be helpful to formally require serious adverse event reporting for medical devices instead of voluntary submission(s).

## 5 Conclusion

This policy brief summarized high-level overview considerations and recommendations for ensuring patient safety and healthcare quality in regulatory oversight frameworks for precision medicine genetic/genomic LDTs in an AI/ML healthcare ecosystem context. Streamlining regulatory systems for clinical sub-specialties and advanced technologies, fostering secure innovation, providing flexibility and efficiency while creating shared value and trust, ensuring transparency and accountability for harms, yields patient safety and healthcare quality. Regardless of the finalized oversight approach selected, the greatest priority at the forefront of policy stakeholder decision-making, must be on safe patient- and family-centered care via access to correct, timely, high-quality diagnosis to protect public trust in science and healthcare.
